# Protocol for a hybrid type I randomized controlled trial evaluating the effectiveness and implementation of a nurse home visiting program for adolescent pregnancy on maternal and infant outcomes

**DOI:** 10.3389/fpsyt.2025.1576428

**Published:** 2025-08-07

**Authors:** Natália Becker, Vinícius Nagy Soares, Débora Tseng Chou, Emilio Abelama Neto, Letícia Aparecida da Silva, Ana Alexandra Caldas Osório, Ariana Gomes Nascimento, Vinnie Marchisio, Alexandro Marcos Menegócio, Adriana Tebaldi Pereira, Andrea Bernardinetti Muller Haas, Alfredo Almeida Pina-Oliveira, Eurípedes Constantino Miguel, Paulo Rossi Menezes, Arthur Caye, Lislaine Aparecida Fracolli

**Affiliations:** ^1^ National Center for Research and Innovation in Mental Health (CISM), São Paulo, Brazil; ^2^ Graduate Program on Human Developmental Sciences, Center for Biological and Health Sciences, Mackenzie Presbyterian University, São Paulo, Brazil; ^3^ Division of Child & Adolescent Psychiatry, Department & Institute of Psychiatry, Faculty of Medicine, University of São Paulo, São Paulo, Brazil; ^4^ Department of Public Health, School of Nursing, University of São Paulo, São Paulo, Brazil; ^5^ Mackenzie Center for Research in Childhood and Adolescence, Center for Biological and Health Sciences, Mackenzie Presbyterian University, São Paulo, Brazil; ^6^ Department of Pediatrics of the Faculty of Medicine, University of São Paulo, São Paulo, Brazil; ^7^ Centro Universitário Max Planck (UniMAX), Indaiatuba, São Paulo, Brazil; ^8^ Centro Universitário de Jaguariúna (UniFAJ), Jaguariúna, São Paulo, Brazil; ^9^ Department of Preventive Medicine, Faculty of Medicine, University of São Paulo, São Paulo, Brazil; ^10^ Graduate Program in Psychiatry and Behavioral Sciences, Universidade Federal do Rio Grande do Sul (UFRGS), Porto Alegre, Brazil

**Keywords:** positive parenting, adolescents, home visits, implementation science, feasibility

## Abstract

**Background:**

Adolescent pregnancy poses significant public health challenges, particularly among vulnerable populations. Nurse home visiting programs, such as *Primeiros Laços*, show promise in improving maternal and child health outcomes, yet their integration into primary healthcare remains underexplored, especially in low-resource settings.

**Aim:**

to evaluate the effectiveness and feasibility of integrating *Primeiros Laços* into primary healthcare services across two municipalities of the state of São Paulo, Brazil.

**Method:**

A hybrid type I randomized controlled trial will be conducted to assess both the clinical effectiveness and implementation feasibility of the program. The study will enroll 200 pregnant adolescents (aged 14–24), who will be randomized into two groups: an intervention group, receiving up to 38 structured nurse home visits from pregnancy until the child’s 24th month, and a control group, receiving standard care. The home visits will focus on promoting maternal and child health, positive parenting practices, and secure mother-infant attachment. Data collection will include validated tools to assess maternal mental health (Alcohol, Smoking, and Substance Involvement Screening Test - ASSIST, Edinburgh Postnatal Depression Scale – EPDS, Tilburg Pregnancy Distress Scale – TPDS, and Generalized Anxiety Disorder-7 - GAD-7), infant development (Infant Behavior Questionnaire – Revised - IBQ-R, Bayley Scales of Infant and Toddler Development - Bayley-III, and Ages and Stages Questionnaire - ASQ-3), mother-infant interactions (Ainsworth’s Maternal Sensitivity Scales, and Maternal Postnatal Attachment Scale), and the quality of the home environment (Infant/Toddler Home Observation for Measurement of the Environment - IT-HOME). Implementation outcomes will be evaluated using the EPIS (Exploration, Preparation, Implementation, Sustainment) framework, semi-structured interviews with key stakeholders (e.g., healthcare providers, program staff, and participants), and the Program Sustainability Assessment Tool to identify factors influencing long-term program viability. Quantitative data will be analyzed using an intention-to-treat approach, while qualitative data will undergo thematic analysis to identify barriers and facilitators to program integration.

**Expected results:**

The intervention is hypothesized to improve maternal and infant outcomes, such as prenatal care adherence, breastfeeding rates, and cognitive and emotional development, while fostering positive parenting practices and secure mother-infant attachment. The implementation analysis will identify key barriers and facilitators to program integration.

**Impact:**

This study will provide evidence on the clinical and practical benefits of nurse home visiting programs for adolescent pregnancy in primary care, guiding their integration, scale-up, and potential adaptation for global maternal and child health initiatives. The findings aim to inform policymakers and healthcare providers on effective strategies to address adolescent pregnancy and improve health outcomes in low-resource settings.

## 1 Introduction

The *World Health Organization* (WHO) defines adolescent pregnancy as gestation in women aged 10 to 19 (1). However, due to behavioral, social, and neurophysiological changes, a recent study suggests extending adolescence to 24 years (2). Hence, public policies could expand social protection benefits to a broader age range, including women aged 20 to 24 as part of the late adolescence group. To prevent early pregnancy, governments have implemented initiatives such as primary health care programs, expanded contraceptive access, and comprehensive sexual education. Although these efforts have contributed to a reduction in adolescent birth rates, early pregnancy continues to affect millions of families worldwide. In 2023, the global adolescent birth rate was 41.3 per 1,000 girls aged 15–19 years (1). In Brazil, 380,000 live births in 2020 - 14% of all births that year were from adolescent mothers, with rates exceeding 21% in economically disadvantaged regions (3).

It is estimated that 55% of unintended adolescent pregnancies end in abortion (1), and that adolescent mothers face higher risks of severe maternal and neonatal complications, including eclampsia, puerperal endometritis, systemic infections, preterm birth, and low birth weight (1). Adolescent and late adolescent pregnancy often perpetuate an intergenerational cycle of poverty, as young mothers are more likely to drop out of school and are excluded from the workforce in order to raise their children (4). This situation is exacerbated by denial of paternity and family conflicts, leading to increased depressive symptoms, stress, anxiety, and even suicidal ideation (5–7). The emotional consequences impair mother-child interactions, which can turn into adverse cognitive, linguistic, emotional, and social development of the child (8).

Nurse home visiting programs are a well-established approach to supporting adolescent mothers and their children. *Primeiros Laços* (First Ties) is a Brazilian nurse home visiting program adapted from models such as *Minding the Baby* (9) and *Nurse Family Partnership* (10). The program was designed to enhance the parenting skills of adolescent mothers, promoting mother-child attachment and child cognitive and emotional development. *Primeiros Laços* was previously tested in its effectiveness in two clinical trials carried out in the city of São Paulo, Brazil (11–14). Fatori et al. (12) found that home visits were associated with improved parenting behaviors, such as increased storytelling and singing to children. Additionally, the intervention group showed better child expressive language, greater maternal responsiveness, and higher rates of secure mother-child attachment (12).

Despite these promising results, the *Primeiros Laços* program was delivered by research assistants rather than local primary health care professionals. This model resulted in a high turnover of visiting professionals and limited engagement from local stakeholders (12, 13). High dropout rates were also observed, which has been extensively observed in studies involving home visits (13, 15, 16). Common reasons for dropout included changes in contact information, substance use disorders and blurred professional boundaries in nurse-adolescent relationships (16). Given these barriers, we hypothesize that integrating the program into primary health care services could improve acceptability and long-term sustainability of the *Primeiros Laços* program. The Exploration, Preparation, Implementation, and Sustainability framework (17, 18) will be applied to inform the systematic adaptation of *Primeiros Laços* and develop a corresponding implementation plan to maximize its adoption and effectiveness in primary health care services. Our study will also apply the Implementation Outcomes Framework proposed by Proctor et al. (19), which offers a taxonomy for implementation outcomes as acceptability, adoption, appropriateness, feasibility, fidelity, penetration, and sustainability aiming to enhance implementation research outcomes. If successful, the program could be scaled up at the national level as part of public health policies to support young mothers and their children. Therefore, this study aims to evaluate the effectiveness and implementation of integrating *Primeiros Laços* into primary health care across two municipalities of the state of São Paulo, Brazil.

### 1.1 Specific objectives

The effectiveness study aims to evaluate the impact of the *Primeiros Laços* program on:

- The psychological symptoms of pregnant adolescents and late adolescents.
- Clinical and health measures for young mothers and children.
- The attachment between young mothers and their children.
- Motor, cognitive, and social development in children.
- Participants’ perceptions of the challenges of motherhood, contributions of home visits, social support networks, exposure to violence, and related topics.

Based on Proctor et al. (19), the implementation study aims to evaluate the following outcomes:

- *Acceptability:* Stakeholder’s perception that the program is satisfactory in its content, complexity and delivery during the preparation, implementation, and sustainability phases.
- *Adoption:* Actions taken by nurses, supervisors, health policy makers, and researchers to integrate the program into primary health care services across the preparation, implementation, and sustainability phases.
- *Feasibility:* Stakeholder’s perception of the program’s usefulness and practicality in the primary health care services during preparation and implementation.
- *Fidelity:* Alignment between the implemented and proposed program during implementation (adherence) and number of visits received by women pregnant/mothers (dosage) (20).
- *Penetration:* Proportion of reach in relation to the total of people invited, considering different stakeholders (services, nurses delivering the program and adolescents), during implementation and sustainability.
- *Sustainability:* The program’s long-term integration and maintenance within primary health care services.

## 2 Materials and methods

### 2.1 Design and samples

This is a type I hybrid randomized controlled trial designed to evaluate the effectiveness and implementation of the *Primeiros Laços* program (21, 22). Studies using this design are essential as it not only evaluates the effectiveness of the intervention but also provides a structured process evaluation. This approach identifies implementation barriers and facilitators, assesses what aspects of the intervention worked or did not work, and determines necessary adaptations for the specific setting (22). Additionally, it helps define the support required for all stakeholders involved in implementation. Data for this implementation-focused analysis can be collected through interviews, surveys, and participant observations, ensuring a comprehensive understanding of the intervention’s real-world applicability. Effectiveness is being investigated in a sample of 200 nulliparous pregnant adolescents and late adolescents (aged between 14 and 24) who are up to 20 weeks pregnant and receiving antenatal care in primary health care services. Participants with severe mental health disorders, psychoactive substance use disorder, or medical conditions that could affect fetal development are excluded from the study. The sample size was determined based on the operational capacity of the primary health care professionals involved in the implementation of the intervention, rather than on a formal *a priori* power calculation. This pragmatic approach reflects the real-world constraints of the study setting, where the objective was to include all eligible participants within the available recruitment period. While this may limit the ability to detect small but clinically meaningful effects, a *post-hoc* power analysis will be conducted to inform the interpretation of the study’s findings. This limitation is acknowledged; however, it aligns with the primary objective of a type I hybrid design, which prioritizes the simultaneous examination of clinical effectiveness and implementation processes. By maximizing the inclusion of eligible participants within real-world service constraints, the study aims to capture a comprehensive picture of both outcomes and contextual factors that influence implementation feasibility, fidelity, and sustainability.

In a stratified 1:1 ratio by health care service, participants are randomly assigned to either an intervention or a control group. The intervention group receives nurse home visits and usual prenatal care in primary health care service, while the control group receives only usual prenatal care provided in the service. Implementation aspects are examined using qualitative and quantitative methods, guided by the Exploration, Preparation, Implementation, and Sustainability (EPIS) framework (18). For convenience, different stakeholders such as managers, nurses, teachers, and researchers take part in the implementation study. Recruiting is taking place between June 2024 and December 2025 and the study will end in September 2028.

### 2.2 Target municipalities

Both Indaiatuba and Jaguariúna are in the interior of São Paulo state, Brazil. Indaiatuba spans 311 km² and has a population of 260,000 residents (23). Between 2019 and 2023, the municipality recorded between 746 and 889 live births annually to mothers aged 14 to 24, accounting for approximately 26% of all births (23). Its healthcare infrastructure includes 17 basic health units staffed by multidisciplinary teams of physicians, nurses, nursing technicians, psychologists, pharmacists, and community health agents. Jaguariúna, in contrast, is a smaller municipality with 59,900 inhabitants across 141.4 km² (23). During the same period, annual births for young mothers ranged from 208 to 243, representing roughly 30% of total births (24). While Jaguariúna’s healthcare teams are similarly composed, key differences exist: the municipality has 11 basic health units, employs fewer community health agents, and only recently implemented the Family Health Strategy program. These structural differences in primary care shape distinct community experiences. Indaiatuba benefits from a well-established network of home visits supported by a robust community health workforce. In contrast, Jaguariúna’s population has had limited exposure to such practices, reflecting its ongoing expansion of primary care outreach. These municipalities were selected due to established partnerships between local governments, sponsors, and the research team. Additionally, implementing this technology in smaller municipalities allows for greater methodological control, facilitating the research’s operationalization. However, as the study is limited to two municipalities with distinct healthcare infrastructures and demographic profiles, the generalizability of the findings to other regions or national contexts may be restricted.

### 2.3 Randomization

In the effectiveness study, a computer-generated algorithm was used to randomly assign primary health care services to either the intervention or control group. Randomization at the service level was chosen to ensure geographical separation between groups, minimizing the risk of sample contamination. In Indaiatuba, 9 services were allocated to the intervention group and 8 to the control group, with a balanced distribution of registered users (52% vs. 48%). In Jaguariúna, 5 services were assigned to the intervention group and 6 to the control group, with a slightly different distribution of users (60% vs. 40%).

### 2.4 Stakeholder engagement

Since the beginning of the research project, stakeholders from both municipalities have been involved in the Exploration and Preparation stages of the EPIS framework. Several meetings were held between the research team and municipal health coordinators, service managers, and nurses. In Jaguariúna, nine primary health care nurses took part in the training for the *Primeiros Laços* program between October and December 2023. In Indaiatuba, 28 professionals were trained between March and April 2024, including primary health care nurses, teachers and students. However, after negotiations with the municipality, it was decided that two nurses contracted by the program would carry out the home visits. As an initial approach, stakeholders and the research team agreed upon these distinct implementation strategies. Both strategies monitor adoption and penetration, aiming to integrate primary health care nurses in the future.

### 2.5 Implementation

The theoretical framework for implementation of the *Primeiros Laços* program follows the EPIS framework (17, 18). Specifically designed for the public service sectors, the EPIS framework outlines the implementation process through four distinct phases: exploration, preparation, implementation, and sustainment. It also identifies key implementation factors—namely, innovation factors and bridging factors—across two contextual levels: the inner and outer contexts. The outer context encompasses external influences such as the service environment and characteristics of the client population, while the inner context pertains to intra-organizational elements, including organizational and provider characteristics. Innovation factors highlight context-specific components that are critical for ensuring the compatibility between an intervention and its implementation setting. Bridging factors serve to connect the inner and outer contexts by addressing elements such as system-level policies, funding mechanisms, partnerships, collaborations, and advocacy efforts. Hence, the model emphasizes: (i) participant acceptability, as engagement and retention are central to the study; (ii) penetration of the intervention; (iii) implementation quality, including protocol fidelity measures; (iv) identification of barriers and facilitators; and (v) assessment of the program’s sustainability. The practical structure of the *Primeiros Laços* program involves four core roles directly responsible for execution, coordinating, and monitoring home visits.

#### 2.5.1 Research team

A multidisciplinary research team plays a pivotal role in implementing, managing, overseeing, and evaluating the program. Their responsibilities include transferring program protocols to municipalities through planning, training professionals, and conducting periodic assessments. The research team holds weekly meetings to monitor the progress of data collection and meets monthly with visiting nurses and supervisors to monitor the adherence of the program. The research team includes a project manager, a general coordinator, associate researchers, PhD students, and research assistants.

#### 2.5.2 Visiting nurse

This is a nursing professional qualified through the *Primeiros Laços* program to conduct home visits for pregnant adolescents and late adolescents. Their work is guided by the program’s theoretical and practical principles, requiring the ability to establish a therapeutic relationship with the participants and provide ongoing support until the child is 24 months old. During visits, the nurse follows a structured protocol employing empowerment strategies and motivational interviewing to engage adolescents and their families. In addition to applying clinical judgment to tailor care to the specific needs of the mother and child, the visiting nurse develops skills across various domains, including environmental health, maternal roles, and support networks. Their practice is grounded in national and local guidelines for nursing, health, and social protection, and all observed concerns are reported to the reflective supervisor.

#### 2.5.3 Reflective supervisor

The reflective supervisor plays a crucial role in supporting and monitoring visiting nurses, ensuring the quality and fidelity (adherence and dosage) of the *Primeiros Laços* program. Their primary function is to provide technical and emotional support to the nurses, assisting them in handling complex situations and helping them find meaning in their work with the mother-baby dyad. Additionally, the reflective supervisor promotes continuous education by organizing courses, workshops, and discussions to enhance nursing practice. Every two weeks, they conduct individual or group supervision sessions and accompany the nurses on home visits when necessary. The supervisor also monitors protocol adherence, reviews records, and collaborates with primary care teams to coordinate responses, particularly for cases requiring mental health follow-up.

#### 2.5.4 Administrative supervisor

This type of supervision involves managing schedules, coordinating leave, allocating resources, and other operational tasks. Most of the time, administrative supervision takes place via email or messaging applications. It is desirable for this professional to know the dynamics of nursing in primary care and to have institutional endorsement from different sectors of the public health service for any decisions that will be made throughout the program. This professional works mainly on logistical and operational challenges.

### 2.6 Training

The theoretical and practical training targeted visiting nurses, supervisors, researchers, and teachers. Equipping professionals affiliated with municipal health departments is critical for effective technology transfer and ensuring the program’s fidelity and sustainability. The training approaches adolescent pregnancy through biological, social and emotional lenses while exploring key concepts such as attachment theory, self-efficacy theory, the bioecological model, social support networks, case studies, and listening and empowerment techniques. Through a 40-hour hybrid course underpinned in Andragogy and Meaningful Learning Theory, built in Modular Object-Oriented Dynamic Learning Environment (Moodle) and available in: https://cursosextensao.usp.br/course/view.php?id=3716. Participants learn to apply the theoretical framework during home visits and accurately complete the structured visitation protocol through live classes with child development and parenting experts, interactive content, forums for support in implementing concepts in local practices, and multiple-choice tests with personalized feedback. Two nurses, specialists with extensive experience in the *Primeiros Laços* Program and university professors, led the in-person meetings for the student’s hands-on activities, evaluation and final certification.

### 2.7 Recruitment process

**Figure 1:**
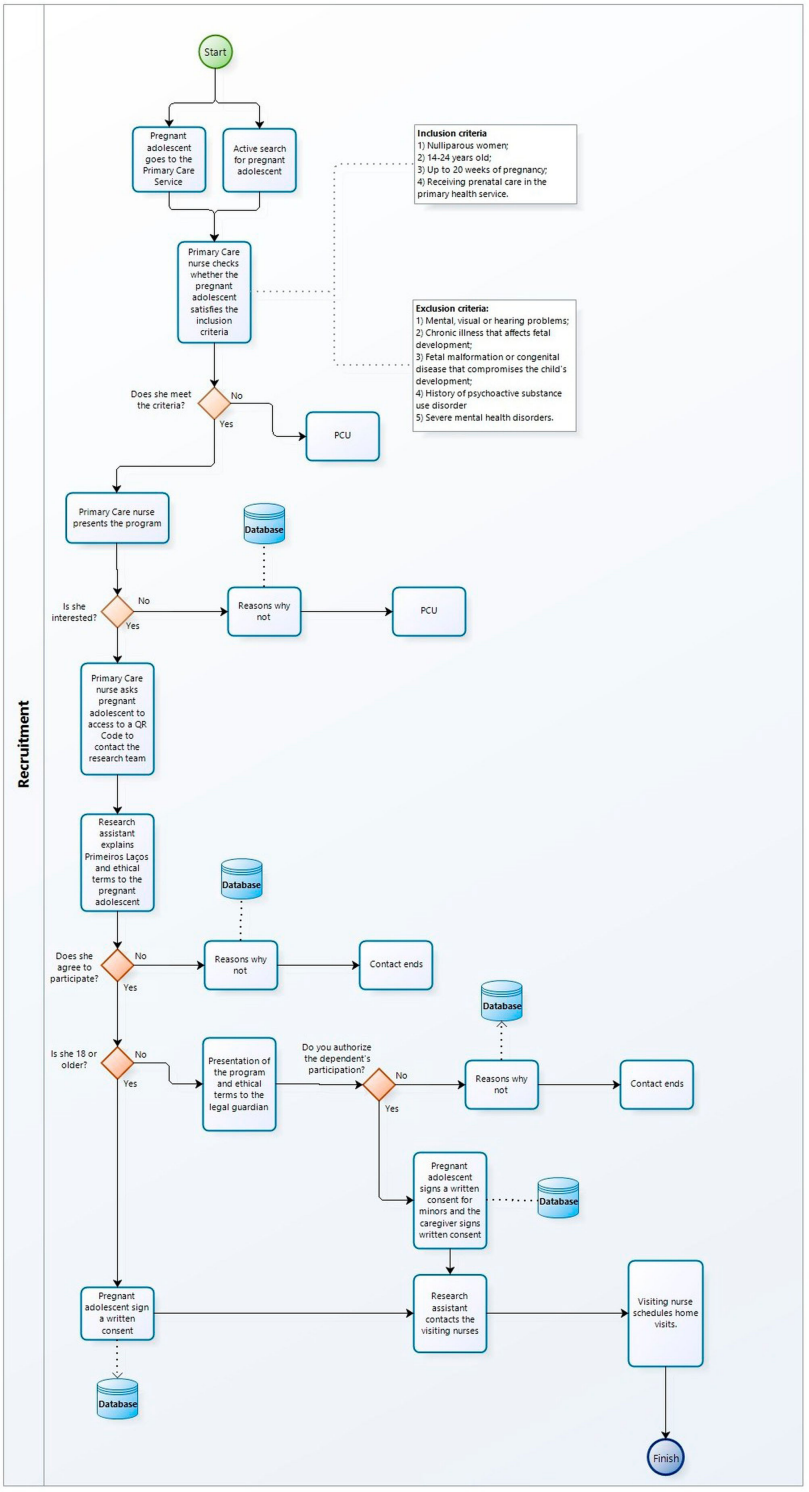
Main stages of the recruitment process.

All pregnant adolescents and late adolescents meeting the selection criteria will be invited to participate. Potential participants are identified by local health care providers and recruited through ethically approved procedures. During the initial prenatal consultation, the nurse introduces the *Primeiros Laços* program using a structured script that outlines its characteristics, potential benefits, duration, home visit topics, and cost-free participation. Interested adolescents and late adolescents access a QR Code to talk to the research team via a messaging application. The research team verifies eligibility, addresses any questions, and explains the ethical terms. Then, a REDCap link is provided for the participant to review and accept the ethical terms and complete the baseline assessments. The consent form also informs participants about randomization and longitudinal data tracking of both maternal and child records up to the child’s second year. Alternatively, the primary health care provider directly invites adolescents and late adolescents, who then provide written consent. Research team members and health care providers were trained in informed consent procedures to minimize coercion and undue influence. Adolescents under 18 years old sign the consent form, and their legal caregivers authorize participation with a separate signature. Those aged 18 to 20 signs independently. An overview of the main stages of the recruitment process is shown in **Figure 1**.

To determine whether eligible pregnant women are being invited to the program, the project manager communicates weekly with primary health care nurses. These nurses provided key details such as the adolescents’ initials, date of birth, and date of last menstrual period. Gradually, the decision of the invited pregnant woman has been classified into “accepted,” “rejected,” “did not respond,” or “expired” — the latter applied if they exceeded 20 weeks of pregnancy or 20 years old.

### 2.8 Intervention group

The intervention group receives 38 home visits delivered by trained nurses carried out between pregnancy and the first 24 months of the infant, distributed as follows: pregnancy (13 biweekly visits), postpartum (3 biweekly visits), 2 to 12 months (12 biweekly visits), and 13 to 24 months of the infant (10 monthly visits). *Primeiros Laços* has a standardized manual, describing all the 38 home visits (**Supplementary Table 1**). It was developed based on three theory frameworks: attachment theory (25), self-efficacy theory (26), and bioecological theory (27). The attachment theory aimed to strengthen the secure and healthy bond between mother and child. The self-efficacy theory sought to empower mothers to make confident decisions regarding their baby’s needs. Meanwhile, the bioecological theory emphasized the importance of considering social, community, and family contexts in parenting. Together, these theories were applied to support young mothers in developing nurturing relationships and providing comprehensive care for their children.

The home visits have a focus on five key areas. *Health and social care* focus on following maternal and child well-being, monitoring development, ensuring proper nutrition, overseeing vaccinations, and promoting accident prevention. *Environmental health* aims to help mothers recognize potential hazards and available resources within their surroundings to create a safer living environment. *Life course* provides guidance to adolescent mothers on career planning, importance of school, and strategies for preventing future pregnancies. *Parenting skills* emphasize the importance of responsive caregiving, fostering secure attachment between mother and child, and stimulating child development. Lastly, *Family and social support* encourages mothers to create supportive social networks and be aware of public services and policy programs.

### 2.9 Control group

The control group receives standard prenatal care through the primary health care service, following national and international guidelines for maternal and child health. High-risk cases will be directed to specialized services within the public health network. This routine care does not involve nurse home visits and includes a recommendation for at least six prenatal consultations throughout pregnancy.

### 2.10 Measures

#### 2.10.1 Effectiveness study

The effectiveness study assesses maternal mental health, parenting behaviors, child development, and the quality of the home environment. Data will be collected at eight time points: baseline (T0), 24 weeks of gestation (T1), 36 weeks of gestation (T2), puerperium (T3), and when the child is 6 months (T4), 12 months (T5), 18 months (T6), and 24 months old (T7). Each instrument will be administered at specific time points, as detailed in **Table 1**. The measurements between T0 and T2 are self-administered and have been collected through a REDCap survey, which was monitored by the project manager. The measurements between T3 and T7 will be conducted by blind evaluators properly trained by the research team, using the REDCap survey.

**Table 1:** Distribution of measurements at different time points (TO to T7).

Measures	Pregnancy			Infant/child				
	TO	T1	T2	T3	T4	T5	T6	T7
Sociodemographic, Clinical, and Health Information	X			X	X	X	X	X
Alcohol, Smoking and Substance Involvement Screening Test (ASSIST)	X							
Brazilian Food Insecurity Scale	X							
Violence Scale adapted from the National Health Survey (2019)	X	X	X					
Edinburgh Postnatal Depression Scale	X	X	X		X	X	X	X
Tilburg Pregnancy Distress Scale (TPDS)	X	X	X					
Generalized Anxiety Disorder - 7 (GAD-7)	X		X					
The Infant Behavior Questionnaire (IBQ)					X	X		X
Ainsworth’s Maternal Sensitivity Scales					X	X		X
Maternal Postnatal Attachment Scale						X		X
Bayley Scale of Infant Development (Bayley)						X		X
Ages and Stages Questionnaire (ASQ)					X	X	X	X
Home Observation for Measurement of the Environment (IT-HOME)					X	X	X	X
Qualitative Interview		X				X		X
Reason for Refusal	X							

Data have been collected at eight time points: baseline (T0), 24 weeks of gestation (T1), 36 weeks of gestation (T2), puerperium (T3), and when the child is 6 months (T4), 12 months (T5), 18 months (T6), and 24 months old (T7).

The symbol x is a "check" for the cell, meaning that the measure in the line will be assessed in the column time.

The list of instruments is as follows:

- Sociodemographic, Clinical, and Health Information: Includes participant details (e.g., name, education, household type, employment, income, and cellphone use). With authorization, medical records may be reviewed for prenatal consultations, test results, clinical events, delivery, postpartum, and pediatric care.
- Alcohol, Smoking, and Substance Involvement Screening Test (ASSIST): WHO-developed questionnaire with eight items assessing the use of nine psychoactive substances. Validated in Brazil, showing good sensitivity and specificity for detecting substance abuse and dependence (28).
- Brazilian Food Insecurity Scale (EBIA): A 14-item scale assessing household food insecurity over the past three months. Validated in Brazil with high internal consistency (α = 0.91–0.94) (29).
- Violence Scale adapted from the National Health Survey (2019): A 12-item questionnaire that assesses different types of violence the respondent may have experienced in the past 12 months. Developed and used in the Brazilian National Health Survey (30).
- Edinburgh Postnatal Depression Scale (EPDS): A 10-item self-report scale assessing postpartum depressive symptoms. Demonstrates good internal consistency (α = 0.87) and excellent discriminatory power (AUC = 0.937). The optimal cutoff (≥10) yields 86.4% sensitivity and 91.1% specificity (31).
- Tilburg Pregnancy Distress Scale (TPDS): A 16-item scale assessing pregnancy-related distress and partner involvement. Responses range from 0 (frequently) to 3 (rarely/never), with total scores from 0 to 48. Factor analysis identified three components: affect and partner involvement, childbirth concerns, and future expectations (32).
- *Generalized Anxiety Disorder-7 (GAD-7):* A seven-item self-report scale measuring anxiety symptoms over the past two weeks. Scores range from 0 to 21, with ≥10 indicating probable generalized anxiety disorder. Validated for Brazilian adolescents (33).
- Infant Behavior Questionnaire – Revised (IBQ-R), Very Short Form (34): A brief, 37-item parent-report measure assessing three broad dimensions of temperament in infants aged 3 to 12 months: Negative Emotionality, Positive Affectivity/Surgency, and Orienting/Regulatory Capacity. Parents rate behaviors observed in the past week on a scale from 1 (never) to 7 (always). The IBQ-R demonstrates high internal consistency (α = 0.71–0.90) and moderate inter-rater agreement (r = 0.42) (34). The *IBQ-R, Very Short Form* was compiled from items translated by Klein, Putnam and Linhares (35) with due authorization from the authors.
- Ainsworth’s Maternal Sensitivity Scales (36): Assesses maternal sensitivity through a filmed nine-minute mother-infant interaction divided into three episodes: i) Free play, where the mother and baby interact with age-appropriate toys; ii) Interaction without toys, where the mother engages the baby using only her presence; and iii) Challenging interaction, where the mother helps the baby play with a slightly difficult toy. Sensitivity is rated on a 1 (“Highly insensitive”) to 9 (“Highly sensitive”) scale, based on the mother’s ability to perceive, interpret, and respond appropriately and promptly to the baby’s signals (36).
- Maternal Postnatal Attachment Scale: Developed by Condon and Corkindale (37) to assess mother-to-infant bonding. It is a 19-item measure, divided into three factors: *Quality of Attachment*, *Absence of Hostility* and *Pleasure in Interaction*. Each item is scored on a 2-, 4- or 5- point scale and lower (versus higher) scores suggest problems in the mother-to-infant bond.
- Bayley Scales of Infant and Toddler Development, Third Edition (Bayley-III): Evaluates five domains of child development: cognition, language, motor skills, socio-emotional development, and adaptive behavior. The first three domains are assessed through direct observation, measuring abilities such as attention, sitting, standing, walking, and sound recognition. The last two domains are evaluated via a caregiver-reported questionnaire. The instrument classifies child development into seven categories, ranging from “extremely low” to “very superior.” In Brazil, its cross-cultural adaptation and psychometric properties were investigated by Madaschi et al. (38).
- Ages and Stages Questionnaire, Third Edition (ASQ-3): Developed by Squires and Bricker (39), the ASQ-3 is a caregiver-administered questionnaire designed to monitor five domains of child development: personal-social, gross motor skills, fine motor skills, problem-solving, and communication. It consists of age-specific questionnaires covering children from 2 to 66 months, with each version containing 30 items (six per domain). Responses are scored as “not yet” (0 points), “sometimes” (5 points), or “yes” (10 points). The ASQ-3 was translated, validated, and adapted for the Brazilian population by Filgueiras et al. (40), demonstrating good psychometric properties (ASQ-BR).
- Infant/Toddler Home Observation for Measurement of the Environment (IT-HOME): Assesses the quality and quantity of stimulation and support available in the child’s home environment and its impact on social, emotional, and cognitive development. The instrument focuses on the child’s experiences rather than the family’s socioeconomic status (41). It consists of an interview with the primary caregiver and direct observation of caregiver-child interactions. The 45 items are divided into six subscales: Emotional and verbal responsiveness of the caregiver, absence of restriction and punishment, organization of the physical and temporal environment and the child’s personal space at home, availability of appropriate materials, toys, and games; caregiver’s involvement with the child, and opportunities for varied daily stimulation. The IT-HOME includes 18 observation items, 24 interview items, and 3 that can be assessed through either method. Each item is scored as 0 (absence) or 1 (presence) based on observations.
- Qualitative Interviews (**Supplementary Table 2**): will be conducted with pregnant women enrolled in the program at three different time points: during pregnancy, at three months postpartum, and at 12 months postpartum. A family member chosen by the participant will also be interviewed. All interviews will be audio-recorded, transcribed anonymously, and analyzed using thematic analysis. *Reason for Refusal:* If an adolescent declines, they may select a reason (e.g., lack of interest, program length). If they prefer not to answer, that option will be available.
- Withdrawal: If the participant expresses a desire to quit the program, the research assistant will ask her the following question via text message: “*For what reason would you like to quit the Primeiros Laços program?*”. The answer will then be sent to the database, and the adolescent will no longer be contacted by the program.

#### 2.10.2 Implementation study

The outcomes of the implementation study are being evaluated at five time points: baseline (T0), and after 6 months (T1), 12 months (T2), 18 months (T3) and 24 months (T4). Except for the qualitative measures, all instruments are self-administered via REDCap survey, and data collection is monitored by the project manager.

- Acceptability: During the preparation phase (EPIS), stakeholders will assess *Primeiros Laços’* complexity and delivery through focus groups conducted by the research team. During implementation, nurses, supervisors, and health coordinators respond to the *Self-assessment tool* (**Supplementary Table 3**) to evaluate the implementation process, including its location and key variables. They will also participate in semi-structured interviews every six months, from the first semester until the program’s conclusion. Additionally, visiting nurses will collect brief participant evaluations at the end of each visit. A 6-month follow-up assessment will be conducted to evaluate sustainability, using the same instruments and stakeholder respondents. Participants will also complete a brief exit interview upon completion of the home visit schedule or in cases of withdrawal. This interview, developed by the research team, will gather insights on the program’s usefulness, perceived benefits for mental and infant health, and overall satisfaction with *Primeiros Laços*. To minimize bias, a research assistant will conduct the interviews.
- Adoption: Actions taken by nurses, supervisors, health policy makers, and researchers to integrate the program into primary health care services during the preparation, implementation, and sustainability phases.
- Feasibility: Stakeholders (health coordinators, supervisors, primary care professionals, and visiting nurses) will assess the program’s usefulness and practicality using the *Program Barriers and Facilitators Questionnaire* (**Supplementary Table 4**).
- Fidelity: Adherence will be evaluated using the *Instrument to Assess Implementation Fidelity* (**Supplementary Table 5**). The alignment between the implemented and proposed program (adherence) and dosage will be monitored for each visit recorded in REDCap. The proportion of visits received by each participant will then be calculated.
- Penetration: The proportion of individuals who engage with the program relative to the total number invited, considering different stakeholders (service providers, nurses delivering the program, and participants), during implementation and sustainability.
- Sustainability: Will be assessed from the start of recruitment (implementation) through the end of the intervention period and the 6-month follow-up. The *Program Sustainability Assessment Tool (PSAT)* (42) will be used to evaluate sustainability, with responses collected from stakeholders (visiting nurses, supervisors, and health coordinators).

The list of instruments is as follows:

- Self-assessment tool for the location and variables of the implementation process (**Supplementary Table 3**): a questionnaire designed by the research team with 15 yes/no items. The tool evaluates the mobilization of Primary Health Care and the clinical content of the actions, aiming to verify whether the premises of the *Primeiros Laços* program are being met.
- Program Barriers and Facilitators Questionnaire (**Supplementary Table 4**): Developed by the researchers, this tool identifies factors that impact program implementation. Health coordinators, supervisors, primary care professionals and visiting nurses rate 37 items across five categories: intervention characteristics, outer context, inner context, individual characteristics and process. Responses use a five-point Likert scale, with an open-ended section for key success factors.
- Instrument to assess implementation fidelity (**Supplementary Table 5**): a tool consisting of 10 items to be evaluated on a Likert scale. It assesses compliance with selection criteria, periodic supervision, adherence to the protocol, and clinical case discussions. Additionally, the instrument includes open-ended fields for stakeholders to highlight the strengths and weaknesses of the implementation, the main challenges faced, and necessary improvements.
- Program Sustainability Assessment Tool (PSAT): A self-administered, 40-item questionnaire designed for public health intervention teams and stakeholders (health managers, researchers). It assesses a program’s sustainability capacity, its ability to maintain activities and benefits overtime helping guide sustainability planning (42). The instrument will be completed by researchers, health managers, and primary care workers involved in the project.
- *Semi-structured Interviews* (**Supplementary Table 3**): will target intervention supervisors, visiting nurses, municipal health managers and researchers, and aim to evaluate acceptability, fidelity (adherence), feasibility and penetration. The interviews were developed by researchers based on existing home visiting programs implementation studies.

### 2.11 Ethical considerations and risk monitoring

The study adheres to the Code of Ethics of the World Medical Association (Declaration of Helsinki). This protocol was reviewed and approved by the Ethical Board Committee of the Faculty of Medicine, University of São Paulo (approval no. 7,253,183, 2024). The study follows the ethical principles outlined in Resolution no. 466/2012 of the National Health Council (43), which establishes guidelines and regulatory standards for research involving human beings in Brazil. It also complies with Law no. 14,874 (44), which specifically governs human research ethics in the country. The study is registered in the Brazilian national system used to manage and oversee research involving human subjects (Plataforma Brasil, n.d.) under CAAE: 74833323.0.0000.0068, ensuring adherence to national regulations for research registration and monitoring.

Participation in the study is entirely voluntary. Participants will receive comprehensive information about the research objectives, methodology, and their rights. To ensure informed consent, the study employs an online Informed Consent Form (ICF) via the REDCap e-consent platform. The ICF is presented in a clear and accessible format, explaining the purpose of the research, the voluntary nature of participation, and guarantees confidentiality and anonymity. Participants will be instructed to read the ICF and confirm their consent by responding to a specific question: “Have you understood the guidelines, and do you agree to participate freely, knowledgeably and spontaneously in this research?” If they agree, they are asked to enter their full name so that it can be attached to their acceptance to take part in the study. Additionally, the participant’s electronic signature will be collected, and a copy of the ICF will be provided via email. The participant will then be directed to a link to the online survey. If they do not agree, the participant will receive a thank-you note, and the contact will be closed. Informed consent will be obtained from all participants, with additional assent from minors and consent from their legal guardians.

The primary safety concerns in this study involve depressive symptoms and suicidal ideation. Risk monitoring follows the guidelines established by Circular Letter No. 13, of June 2, 2020 (45), which regulates the processing of Adverse Events in Brazil, and is aligned with the provisions of Law No. 14,874. The clinical trial’s progress and participant safety are being monitored by the visiting nurses’ supervisor and project manager. Researchers are overseeing participant safety based on depression severity and suicide risk assessments from home visits or periodic evaluations. If symptoms worsen or suicidal thoughts emerge, primary health care services are notified to take appropriate action, following the Adverse Effects Protocol.

Adverse Events in the study refer to the occurrence or worsening of depressive symptoms, assessed using the Edinburgh Postnatal Depression Scale, while Serious Adverse Events include hospitalization, participant or child death, suicide attempts, or any other unforeseen event related to the study. Although the intervention aims to improve mental health outcomes for adolescent pregnant women, symptoms may still worsen. Adverse Events are identified through home visits and data collection and are reported to primary health care services for appropriate follow-up. Serious Adverse Events must be communicated within specific timeframes, with deaths reported within 72 hours. The project manager and coordinator oversee the review and documentation of these events, ensuring timely reporting to the ethics committee. Due to the nature of the intervention, study interruption is not anticipated, and decisions are made collaboratively by the research team.

Data security

Only the project manager, project coordinator and the researcher responsible for ethical issues have broad access to REDCap tools. Nevertheless, editing in the database is not available and must be requested from the data center (composed of independent professionals) with reasonable justification. Visiting nurses, supervisors, and research assistants have limited access to identifiable data necessary for their routine tasks. Evaluators of the effectiveness measures do not have access to any personal information capable of identifying the participant’s group. The project manager is responsible for monitoring procedures during the study, as outlined in the protocol and operations manual. With the participant’s authorization, personal information can be shared with primary health care professionals and members of the research team. If the participant is in emotional distress and the team is unable to contact her, family members can be notified. Access to REDCap is protected by username and password and any changes made are saved in metadata.

### 2.12 Statistical Analysis

The analyses will follow the intention-to-treat (ITT) approach. Given the known risk of attrition in home-visiting trials—often due to participant mobility or disengagement—this approach helps minimize bias by preserving the initial group allocation regardless of adherence to the intervention. Descriptive statistics will be used to characterize the variables in the dataset. Normality, homogeneity of variances, and sphericity will be assessed using the Shapiro-Wilk, Levene, and Mauchly tests, respectively. Given the nature of the variables analyzed, many outcomes are expected to deviate from a normal distribution, requiring the use of statistical models better suited to alternative distributions. In this sense, to assess the effect of the group (intervention vs. control), time (repeated measures), and interaction (group*time) on continuous outcomes, Generalized Mixed Models will be employed. These models allow for the analysis of unbalanced data, account for correlations in repeated measures over time, and are robust to missing data under the assumption of Missing at Random. Participants will be included as a random effect, given the expected high interindividual variability. Model fit will be evaluated by comparing different outcome distributions using the Akaike Information Criterion (AIC). Additionally, the distribution of residuals will be inspected through Q-Q plots to identify potential deviations from model assumptions. If substantial biases or violations are detected, sensitivity analyses will be conducted to test the robustness of the findings. A 5% significance level will be adopted for all analyses. The qualitative measures will adhere to the Consolidated Criteria for Reporting Qualitative Research (COREQ) to ensure methodological rigor and transparency and will be analyzed using thematic analysis.

## 3 Expected results

For primary outcomes, we expect the *Primeiros Laços* program to demonstrate measurable benefits across multiple domains. During pregnancy, a higher proportion of participants in the intervention group is expected to achieve the minimum recommended prenatal care attendance (≥6 appointments). At 6, 12, 18, and 24 months postpartum, infants in the intervention group are anticipated to show better overall development on the ASQ compared to those in the control group. Additionally, we expect higher exclusive breastfeeding rates during the first six months and fewer hospitalizations due to respiratory infections at 12 and 18 months. By 24 months postpartum, a lower proportion of new pregnancies with intervals shorter than 24 months is projected in the intervention group. Assessments of home environment quality and positive parenting, measured by IT-HOME at 6, 12, 18, and 24 months, are also expected to yield higher scores in the intervention group.

For secondary outcomes, we anticipate reductions in the incidence of low birthweight, maternal stress, depressive and anxiety symptoms during and after pregnancy, and maternal exposure to violence. Additionally, we expect improvements in child neurodevelopment, as measured by the Bayley Scales, and in secure caregiver attachment, assessed using Ainsworth’s Maternal Sensitivity Scales. Lower maternal school dropout rates by the child’s first year are also anticipated.

The implementation evaluation will focus on stakeholder perspectives (pregnant women/mothers, visiting nurses, supervisors, and municipal health managers) and key program performance metrics. Data collected via REDCap will assess program penetration (proportion of eligible participants invited and enrolled), dosage (number of home visits received by pregnant women/mothers). These findings will contribute to the refinement and potential scalability of the *Primeiros Laços* program.

## 4 Discussion

Young mothers and their babies may benefit from nurse home visiting programs, such as *Primeiros Laços*. However, these initiatives have mostly been tested in controlled experimental trials. Here, we developed a protocol to evaluate the effectiveness and implementation of this intervention within primary health care services in two municipalities in São Paulo. This hybrid type-I randomized controlled trial integrates nurses from primary care as program facilitators, allowing us to assess whether the outcomes observed in previous trials are replicated in real-world conditions. Additionally, the study aims to identify barriers and facilitators to implementing home visiting programs through the Brazilian public health system.

The study protocol was carefully designed based on prior trials (11–14) and in collaboration with multiple stakeholders, including local health professionals, policymakers, national and international experts in nursing home visiting programs, researchers in implementation science, and specialists in adolescent pregnancy and early childhood development. As a first result, the implementation process revealed institutional and operational challenges that affected feasibility within primary care. From the outset, we anticipated difficulties in fidelity, considering the multiple demands for primary health care services.

The use of a hybrid design offers several advantages, particularly because evaluating implementation outcomes alongside effectiveness is crucial to understanding the true impact of an intervention (20). Implementation outcomes not only help determine whether the relationship between an intervention and its primary and secondary outcomes is influenced by implementation quality but also prevent potentially misleading conclusions about its effectiveness. High implementation fidelity strengthens confidence that observed effects are directly attributable to the program. Additionally, it ensures that professionals are correctly implementing the intervention and enables a more reliable synthesis of findings in secondary research. This is particularly relevant in real-world settings, where greater variability in implementation is expected compared to controlled experimental conditions.

In this study, we will examine the relationship between implementation outcomes—such as dose, adherence, feasibility, and penetration—and key outcome measures, including attachment between young mothers and their children, positive parenting skills, and children’s motor, cognitive, and social development. The expectation is that higher fidelity and sustainability in program delivery will be associated with improved scores, particularly in these domains. By integrating effectiveness and implementation analyses, this study aims to provide a comprehensive understanding of both the clinical impact and real-world feasibility of *Primeiros Laços*, contributing to the refinement and scalability of nurse home visiting programs within primary healthcare services.

This protocol also highlights the importance of careful planning and collaborative development when introducing innovative programs in primary health care services. Identifying both barriers and facilitators allows for strategic adaptations that increase the likelihood of success and maximize benefits for the target population. Sustainability and potential expansion will depend on the ability to adjust to local contexts and strengthen enabling factors. Finally, we emphasize that this study is expected to provide compelling evidence on the clinical effectiveness and practical feasibility of a nurse home visiting program for adolescent pregnancy delivered in primary care services. The results should help shape policy and practice by guiding the integration and expansion of these services, ultimately bridging care gaps for vulnerable young mothers and their children and offering a replicable model for similar initiatives worldwide.

Despite the strengths of this protocol, some limitations must be acknowledged. First, the sample size was not determined based on a formal *a priori* power calculation but was instead defined by the operational capacity of the primary health care services involved. While this pragmatic choice reflects real-world constraints and aligns with the hybrid type I design—which prioritizes implementation alongside effectiveness—it may limit the ability to detect small but clinically meaningful effects. Second, the study is conducted in only two municipalities in São Paulo state, each with distinct healthcare infrastructures. Although this allows for the assessment of implementation across different service contexts, it may restrict the generalizability of the findings to other regions or national settings. Third, as commonly observed in home-visiting programs, participant attrition poses a potential threat to internal validity, particularly given the mobility and socioeconomic vulnerability of the target population. While the use of an intention-to-treat (ITT) analysis mitigates some bias, differential dropout across groups could still impact the interpretation of results. These limitations are important considerations for future studies and will be carefully addressed in the interpretation of findings.
