# Supplementary material for: Protocol for a hybrid type I randomized controlled trial evaluating the effectiveness and implementation of a nurse home visiting program for adolescent pregnancy on maternal and infant outcomes
Source: Front Psychiatry. 2025 Aug 7;16:1576428. doi: 10.3389/fpsyt.2025.1576428 (PMC12367722; doi:10.3389/fpsyt.2025.1576428)
Supplement: Supplementary file 1 [file Table1.docx]

Supplemental 1. Topics to be covered in home visits.

| Visit | Stage | Topics |
| --- | --- | --- |
| 1 | Pregnancy | Introduction visit |
| 2 | Pregnancy | Physical health |
| 3 | Pregnancy | Social network |
| 4 | Pregnancy | Physical health |
| 5 | Pregnancy | Bonding and Attachment |
| 6 | Pregnancy | Family, friends, home, and community |
| 7 | Pregnancy | Social network |
| 8 | Pregnancy | Bonding and Attachment |
| 9 | Pregnancy | Physical health |
| 10 | Pregnancy | Bonding/Attachment |
| 11 | Pregnancy | Physical health and life plan |
| 12 | Pregnancy | Physical health and social network |
| 13 | Pregnancy | Bonding and Attachment |
| 14 | Postpartum | Physical health |
| 15 | Postpartum | Parenting |
| 16 | Postpartum | Supportive social network |
| 17 | 2 to 12 months | Health and breastfeeding |
| 18 | 2 to 12 months | Bonding and Attachment |
| 19 | 2 to 12 months | Sexual and reproductive health |
| 20 | 2 to 12 months | Child growth and development |
| 21 | 2 to 12 months | Immunization |
| 22 | 2 to 12 months | Home environment and safety |
| 23 | 2 to 12 months | Socialization and play |
| 24 | 2 to 12 months | Parenting styles |
| 25 | 2 to 12 months | Child development |
| 26 | 2 to 12 months | Introduction to solid foods |
| 27 | 2 to 12 months | Work and school |
| 28 | 2 to 12 months | Daycare and Primary Healthcare Unit |
| 29 | 13 to 24 months | Play |
| 30 | 13 to 24 months | Child development and growth |
| 31 | 13 to 24 months | Nutrition |
| 32 | 13 to 24 months | Maternal involvement |
| 33 | 13 to 24 months | Safety |
| 34 | 13 to 24 months | Child development and growth |
| 35 | 13 to 24 months | Non-violent communication |
| 36 | 13 to 24 months | Non-violent communication |
| 37 | 13 to 24 months | Life project |
| 38 | 13 to 24 months | Child protection network |
