# Supplementary material for: Protocol for a hybrid type I randomized controlled trial evaluating the effectiveness and implementation of a nurse home visiting program for adolescent pregnancy on maternal and infant outcomes
Source: Front Psychiatry. 2025 Aug 7;16:1576428. doi: 10.3389/fpsyt.2025.1576428 (PMC12367722; doi:10.3389/fpsyt.2025.1576428)
Supplement: Supplementary file 2 [file Table2.docx]

**Supplemental 2**

**Interview Guide for Qualitative Evaluations of Primeiros Laços (First Ties) Program**

**Group 1 - Adolescents (during pregnancy)**

### **Introduction**

Introduction of the interviewer, presentation of the research, information about confidentiality, and oral consent for the interview and recording.

- How old are you?
- How long have you been followed by the PL program?
- How far along are you in your pregnancy?
- Who do you live with?
- Do you study or work?

### **A) Pregnancy**

- How has your experience with pregnancy been?
  (Leave this question very open to hear the first things that come to mind, as a warm-up.)
- What has changed the most in your life after discovering that you are pregnant?
  (Routine, mindset, concerns, desires.)
- Have you noticed any changes in your family since you became pregnant? If so, what changes?
  (Relationship with parents, partner.)
- Have you noticed any changes in your other social relationships?
  (Friends, school)
- How has it been for you to experience the changes in your body during pregnancy?
- Do you have any concerns about facing challenges in motherhood? If so, what are they?
  (Time management, financial issues, feelings of incapacity, fears.)
- Would you consider that you have a support network? What kind?
  (Partner, family, friends, church, public resources.)

### **B) Primeiros Laços Program**

- What are your thoughts on participating in the Primeiros Laços (PL) program?
  (General impressions about the program.)
- Is there anything you think could be improved in the program to make it better?
  (Topics covered, relationship with the visiting professional, number of visits.)
- Do you think the program can bring any changes to your life as a mother?
  (Skill development, change in perspectives, increased confidence.)
- Do you think the PL program has influenced your relationship with healthcare services?
  (Greater adherence to prenatal care, increased contact with health centers, awareness of available resources.)

*Thank you for your responses so far; they have been very helpful to us. Now, I would like to ask about some topics that might be a bit more personal. Please feel free to skip any questions you do not wish to answer. This will not affect the interview in any way or impact your relationship with healthcare services.*

### **C) Violence**

- Regarding how you want to raise your child, is there anything your parents did with you that you would like to do similarly?
- And is there anything you would like to do differently?
  (Here, pay attention to issues related to violence, and if the participant is open, explore the topic further.)

### **D) Closing Questions**

- Is there anything else you would like to share about the topics we discussed?
- We would like to hear your opinion about this conversation. How was it for you to answer these questions? What do you think about them?

**Group 2 - Adolescents (3 months and 12 months postpartum)**

### How long have you been a part of the program?

### **A) Motherhood**

- How has your experience with motherhood been ?
  (Previous planning, bond with the baby; identity issues within the social context, transition from adolescence to womanhood, perception of body changes.)
- Are you facing or do you fear facing any challenges in motherhood? If so, what are they?
  (Time management, financial issues, feelings of incapacity, fears.)
- What has changed the most in your life after becoming a mother?
  (Routine, mindset, concerns, desires.)
- Would you consider that you have a support network?
  What kind?
  (Partner, family, friends, church, public resources.)

### **B) Primeiros Laços Program**

- What are your thoughts on participating in the Primeiros Laços (PL) program?
  (General impressions about the program.)
- Is there anything you think could be improved in the program to make it better?
  (Topics covered, relationship with the visiting professional, number of visits, areas for improvement.)
- Do you think the program has brought any changes to your life as a mother?
  (Taught new skills, changed perspectives, increased confidence.)
- Do you think the PL program influenced your relationship with healthcare services?
  (Greater adherence to pediatric care, increased contact with health centers, awareness of available resources.)

### **C) Violence**

- Regarding how you want to raise your child, is there anything your parents did with you that you are trying to do similarly?
- And is there anything you are trying to do differently?

**D) Closing Questions**

- Is there anything else you would like to share about the topics we discussed?
- How did you feel about this interview?

**Group 3 - Nurses, Supervisors, Project Managers and Researchers**

### **For All**

### Could you describe your current role in the project?

### What are your impressions of the current state of the Primeiros Laços implementation process? (Initial general impressions)

### **For Nurses**

- What did you think of the Primeiros Laços training program? (Did the nurses feel confident in carrying out the implementation?)
- What are your impressions of the home visit protocols? (Do they help? Are they clear and understandable?)
- During the training, did you conduct any home visits? If so, how was your visit?
  (Who participated, family receptivity, adolescent’s interest, visit duration, difficulty in applying the protocol.)
- How has the contact with the visited families been?
  (Was there good receptivity? Did the families feel comfortable welcoming a professional into their home?)
- How has the supervision of visiting nurses been so far?

### **For All**

- What do you think will be the biggest challenges in implementing the program?
- Do you think the program is feasible for Primary Care?
- If you had to improve the program, what changes would you make?
- Do you perceive any kind of resistance from pregnant teenagers?
- Do you perceive any kind of resistance from Primary Care professionals?
- Is there any additional support you would like to receive?

#### What are your expectations for the next steps?
