# Supplementary material for: Protocol for a hybrid type I randomized controlled trial evaluating the effectiveness and implementation of a nurse home visiting program for adolescent pregnancy on maternal and infant outcomes
Source: Front Psychiatry. 2025 Aug 7;16:1576428. doi: 10.3389/fpsyt.2025.1576428 (PMC12367722; doi:10.3389/fpsyt.2025.1576428)
Supplement: Supplementary file 3 [file Table3.docx]

**Supplemental 3**

**SELF-ASSESSMENT TOOL FOR THE SITE AND IMPLEMENTATION PROCESS VARIABLES**

**Indicate which elements of the Primeiros Laços program are implemented in your service.**

|  | **Yes** | **No** |
| --- | --- | --- |
| **Implementation Process** | | |
| At the Primary Healthcare Service, care is provided by an interdisciplinary team, including a psychologist and a home visiting nurse explicitly trained in the Primeiros Laços program model. |  |  |
| At the Primary Healthcare Service, first-time mothers are prioritized and enrolled in prenatal care between 16 and 20 weeks of pregnancy. |  |  |
| Primeiros Laços interventions are delivered at home through appropriately timed home visits, beginning during pregnancy, with research and implementation teams, and include mental health care when necessary. |  |  |
| Parents enrolled in Primeiros Laços are assessed for life-threatening medical conditions, psychosis, or severe substance abuse. Families with these complex needs are excluded from Primeiros Laços and referred to alternative services. |  |  |
| **Clinical Content** | | |
| Primeiros Laços nurses or visiting professionals focus on enhancing health, mental health, parent-child relationships, attachment, child development, parental support, and life-course outcomes with families. |  |  |
| Primeiros Laços nurses or visiting professionals coordinate with healthcare professionals to promote child and parental health and safety, providing mental health assessments and therapeutic services at home if needed. |  |  |
| Primeiros Laços nurses or visiting professionals integrate parenting into direct care, aiming to enhance parental caregiving capacities and encourage reflection on thoughts and emotions as a means of better understanding them. |  |  |
| Primeiros Laços nurses or visiting professionals and supervisors regularly engage in reflective supervision. |  |  |
| Primeiros Laços nurses or visiting professionals work intergenerationally to foster supportive and caring relationships, as well as the development of secure attachment and regulatory capacities. |  |  |
| Primeiros Laços nurses or visiting professionals focus on developing the visitor-parent relationship as the primary agent of change. |  |  |
| Nurses or visiting professionals work in partnership with families to promote shared decision-making with a non-judgmental stance and a strengths-based perspective. |  |  |
| All members of the Primary Healthcare Unit team emphasize cultural awareness and respect for diverse family structures through diversity-informed practices. |  |  |
| There is a focus on prevention through a trauma-informed approach, defined as fostering resource development to buffer the impacts of trauma, adverse childhood experiences, and toxic stress on health and mental health. |  |  |
| A flexible curriculum is used, based on Primeiros Laços training, manuals, and the theories underlying the model, but adapted to meet the individual needs of families. |  |  |
| Supervision is provided as described and defined in Primeiros Laços training, including supporting nurses/visiting professionals in maintaining a reflective stance, establishing a warm and non-judgmental relationship with families, and promoting self-care. Primeiros Laços nurses or visiting professionals and supervisors regularly engage in reflective supervision. |  |  |
