# Supplementary material for: Protocol for a hybrid type I randomized controlled trial evaluating the effectiveness and implementation of a nurse home visiting program for adolescent pregnancy on maternal and infant outcomes
Source: Front Psychiatry. 2025 Aug 7;16:1576428. doi: 10.3389/fpsyt.2025.1576428 (PMC12367722; doi:10.3389/fpsyt.2025.1576428)
Supplement: Supplementary file 4 [file Table4.docx]

**Supplemental 4**

**Barriers and Facilitators to the Implementation of Primeiros Laços**

**Implementation Factors**

Dear colleague, we request your collaboration in completing the questionnaire below, which serves as an interview guide for personnel involved in the implementation of the Primeiros Laços program. This guide aims to understand the organizational factors that may act as barriers or facilitators in the implementation of the program.

The following items describe various organizational factors that may potentially affect the implementation of Primeiros Laços in your unit. This instrument aims to identify the level of importance you believe each factor will have in the success or failure of the program's implementation.

Source of the intervention

How important is it for the implementation of Primeiros Laços that the initiative is developed externally or internally?No importance

Little importance

Moderate importance

Important

Very important

Strength and quality of evidence

How important is it for the implementation of Primeiros Laços that scientific evidence, clinical experience, or local experiences support the belief that the desired outcomes will be achieved?

No importance

Little importance

Moderate importance

Important

Very important

Relative advantage

How important is it for the implementation of Primeiros Laços that stakeholders perceive the program as advantageous compared to other approaches aimed at caring for adolescent mothers?

No importance

Little importance

Moderate importance

Important

Very important

Adaptability

How important is the degree of adaptation, adjustment, or refinement of the Primeiros Laços program to meet local needs?

No importance

Little importance

Moderate importance

Important

Very important

Testability

How important is the ability to test Primeiros Laços on a small scale and to reverse course (undo implementation) if necessary?

No importance

Little importance

Moderate importance

Important

Very important

Complexity

How important is the perceived complexity of implementing Primeiros Laços, as reflected in its duration, scope, radicality, disruption, complexity, and the number of steps required for implementation?

No importance

Little importance

Moderate importance

Important

Very important

Project quality and packaging

How important is the perceived excellence in the way your initiative is designed and disseminated as a program for the implementation of Primeiros Laços?No importance

Little importance

Moderate importance

Important

Very important

Cost

How important are the execution costs of Primeiros Laços, as well as the initial costs associated with implementation, including time and effort?

No importance

Little importance

Moderate importance

Important

Very important

**Factors related to the external environment**

Patient needs and resources

How important is it for the implementation of Primeiros Laços that the needs of adolescent mothers, as well as the barriers and facilitators to meeting those needs, are accurately understood and prioritized by the involved institutions?

No importance

Little importance

Moderate importance

Important

Very important

Cosmopolitanism

How important is it for the implementation of Primeiros Laços that the involved institutions are networked with other external organizations related to maternal and child care and mental health?

No importance

Little importance

Moderate importance

Important

Very important

Peer pressure

How important is the mimetic or competitive pressure to implement Primeiros Laços, typically because most or other key peer or competitor organizations have already implemented it or are seeking a competitive advantage?No importance

Little importance

Moderate importance

Important

Very important

External Policies and Incentives

How important are external strategies for promoting the implementation of Primeiros Laços, including policies and regulations (governmental or from another central entity), recommendations and guidelines, pay-for-performance/performance measures, collaborations, and public or benchmark reports?

No importance

Little importance

Moderate importance

Important

Very important

Structural Characteristics

How important are the social positioning, maturity, and size of the involved institutions for the implementation of Primeiros Laços?

No importance

Little importance

Moderate importance

Important

Very important

Factors Related to the Internal Environment

Networks and Communication

How important are the nature and quality of social networks, as well as the nature and quality of formal and informal communications within the involved institutions for the implementation of Primeiros Laços?

No importance

Little importance

Moderate importance

Important

Very important

Culture

How important are the norms, values, and basic assumptions of the involved institutions for the implementation of Primeiros Laços?

No importance

Little importance

Moderate importance

Important

Very important

Tension for Change

How important is the degree to which participants perceive the current state of care for adolescent mothers and their children as inadequate or in need of change for the implementation of Primeiros Laços?

No importance

Little importance

Moderate importance

Important

Very important

Compatibility

How important is the degree of alignment between the institutional meanings and values and the Primeiros Laços initiative, as well as how well they fit into existing workflows and systems in Primary Health Care?

No importance

Little importance

Moderate importance

Important

Very important

Relative Priority

How important is the shared perception among stakeholders regarding the relevance of the program in the involved institutions for the implementation of Primeiros Laços?

No importance

Little importance

Moderate importance

Important

Very important

Organizational Incentives and Rewards

How important are incentives such as goal-sharing awards, performance evaluations, promotions, and salary increases, as well as less tangible incentives such as increased respect, in promoting Primeiros Laços?

No importance

Little importance

Moderate importance

Important

Very important

Goals and Feedback

How important is it for the implementation of Primeiros Laços that the program’s goals are clearly communicated, put into practice, and fed back to the team, ensuring alignment with established objectives?

No importance

Little importance

Moderate importance

Important

Very important

Learning Climate

How important is a climate in which:

a) Leaders express their own fallibility and the need for assistance and contributions from team members;

No importance

Little importance

Moderate importance

Important

Very important

b) Team members feel they are essential partners, valued, and knowledgeable about the change process;

No importance

Little importance

Moderate importance

Important

Very important

c) Individuals feel psychologically safe to experiment with new methods;

No importance

Little importance

Moderate importance

Important

Very important

d) There is sufficient time and space for reflective thinking and evaluation?

No importance

Little importance

Moderate importance

Important

Very important

Leadership Engagement

How important are the commitment, involvement, and responsibility of leaders and managers in implementing the Primeiros Laços initiative?

No importance

Little importance

Moderate importance

Important

Very important

Available Resources

How important are the level of resources dedicated to implementation and ongoing operations, including funding, training, education, physical space, and time, for the implementation of Primeiros Laços?

No importance

Little importance

Moderate importance

Important

Very important

Access to Knowledge and Information

How important is the ease of access to clear information and knowledge about Primeiros Laços and how to incorporate it into work in healthcare and/or education?

No importance

Little importance

Moderate importance

Important

Very important

**Characteristics of Individuals**

Knowledge and Beliefs About the Initiative

How important are the attitudes of those involved in relation to the intervention, the value attributed to it, and familiarity with facts, values, and principles related to the program for the implementation of Primeiros Laços?

No importance

Little importance

Moderate importance

Important

Very important

Self-Efficacy

How important is the belief of those involved in their own ability to perform the actions that comprise the Primeiros Laços program?

No importance

Little importance

Moderate importance

Important

Very important

Individual Stage of Change

How important is the characterization of the phase (e.g., pre-contemplation or contemplation) in which the participants are, as they progress toward skilled, enthusiastic, and sustained use of Primeiros Laços?

No importance

Little importance

Moderate importance

Important

Very important

Individual Identification with the Organization

How important is the way individuals perceive the organizational structure, their relationship to it, and their level of commitment to this structure for the implementation of Primeiros Laços?

No importance

Little importance

Moderate importance

Important

Very important

Other Personal Attributes

How important are other personal characteristics of participants, such as tolerance for differences, intellectual ability, motivation, values, competence, capacity, and learning style for the implementation of Primeiros Laços?

No importance

Little importance

Moderate importance

Important

Very important

**Process**

Planning

How important is the extent to which planning for the implementation of the initiative is developed in advance and the quality of that planning for the implementation of Primeiros Laços?

No importance

Little importance

Moderate importance

Important

Very important

Opinion Leaders

How important are the individuals within participating institutions who have formal or informal influence over the attitudes and beliefs of their colleagues regarding the implementation of Primeiros Laços?

No importance

Little importance

Moderate importance

Important

Very important

Formally Designated Implementation Leaders

How important are the individuals in participating institutions who have been formally designated as responsible for implementing the program, such as coordinators, project managers, team leaders, or similar roles?

No importance

Little importance

Moderate importance

Important

Very important

Key Individuals

How important are the individuals dedicated to supporting, promoting, and advancing the Primeiros Laços program, overcoming indifference or resistance within institutions?

No importance

Little importance

Moderate importance

Important

Very important

External Change Agents

How important are individuals affiliated with an external entity who formally influence or facilitate the implementation of the Primeiros Laços program?

No importance

Little importance

Moderate importance

Important

Very important

Key Stakeholders

How important is the inclusion of stakeholders in the development and implementation of the Primeiros Laços program? (Note: A stakeholder is someone who can affect or be affected by the program.)

No importance

Little importance

Moderate importance

Important

Very important

Execution

How important is it to carry out or implement the Primeiros Laços program according to the planned framework?

No importance

Little importance

Moderate importance

Important

Very important

Reflection and Evaluation

How important are quantitative and qualitative feedback on the progress and quality of implementation, accompanied by regular individual and team analyses of progress and overall experience quality, for the implementation of Primeiros Laços?

No importance

Little importance

Moderate importance

Important

Very important

**Final Questions**

What are the three factors you believe will most determine the success of your initiative?

If you have any comments, add them here:
