# Supplementary material for: Protocol for a hybrid type I randomized controlled trial evaluating the effectiveness and implementation of a nurse home visiting program for adolescent pregnancy on maternal and infant outcomes
Source: Front Psychiatry. 2025 Aug 7;16:1576428. doi: 10.3389/fpsyt.2025.1576428 (PMC12367722; doi:10.3389/fpsyt.2025.1576428)
Supplement: Supplementary file 5 [file Table5.docx]

**Supplemental 5**

**INSTRUMENT FOR VERIFYING THE FIDELITY OF THE PRIMEIROS LAÇOS IMPLEMENTATION PROCESS**

**For each question, select the most appropriate response for families enrolled in Primeiros Laços.**

| **Questions** | **Never** | **Sometimes** | **Always** |
| --- | --- | --- | --- |
| 1. Do they voluntarily participate in the program? |  |  |  |
| 2. Do they have the same nurse or visiting professional throughout the intervention? |  |  |  |
| 3. Did they receive at least five home visits during prenatal care? |  |  |  |
| 4. Are they first-time mothers/fathers? |  |  |  |

Please evaluate the following items:

| **In your opinion, how is:** | **Very poor** | **Poor** | **Fair** | **Good** | **Very good** |
| --- | --- | --- | --- | --- | --- |
| 1. The effectiveness of local supervisors in balancing administrative, clinical, and reflective supervision? |  |  |  |  |  |
| 2. The level of adherence to Primeiros Laços requirements, including continuous and careful review of protocol and clinical procedure compliance (such as the use of clinical and/or research forms and regular review of procedures, approaches, competencies, and self-assessment checklists for clinicians and supervisors)? |  |  |  |  |  |

3. Please, comment your answeres:

__________________________________________________________________________________________________________________________________________________________________________________________________________________________________________________________________________________________________________________________________________________________________________________________________________________________________________________________________________________________________________________________________________________________________________________________________________________________________________________________________________________________________________________________________________________________________________________________________________________________________________________________________________________________________________________________________________________________________________________________________________________________________________________________________________________________

Please indicate the frequency of the following questions:

| **Questions** | **Never** | **Sometimes** | **Always** |
| --- | --- | --- | --- |
| 11. Are Primeiros Laços team meetings (in-person or via video conference), including case discussions, held regularly (preferably weekly)? |  |  |  |
| 12. Do all Primeiros Laços team members (including supervisors and visitors) regularly attend and participate in team meetings? |  |  |  |
| 13. Do visitors receive regular supervision (preferably weekly) with local coordinators or Primeiros Laços supervisors? |  |  |  |
| 14. Are high-quality case presentations and in-depth case discussions incorporated into team meetings, supervision sessions, and consultations with the Primeiros Laços central team? |  |  |  |

15. Please, comment your answeres:

____________________________________________________________________________________________________________________________________________________________________________________________________________________________________________________________________________________________________________________________________________________________________________________________________________________________________________________________________________________________________________________________________________________________________________________________________________________________________________________________________________________________________________________________________________________________________________________________________________________________________________________________________________________________________________________________________________________________________________________________________________________

16. From your Primeiros Laços team’s perspective, what is going well with the implementation, and what was the most useful part of the training or home visit protocols? Important points to comment on: whether there was a good initial connection with the family and whether internal and external supervision is effective.

__________________________________________________________________________________________________________________________________________________________________________________________________________________________________________________________________________________________________________________________________________________________________________________________________________________________________________________________________________________________________________________________________________________________________________________________________________________________________________________________________________________________________________________________________________________________________________________________________

17. What was the most challenging aspect of implementation? Where is there room for growth or improvement in implementation? How was the adaptation of the model to the local context?

__________________________________________________________________________________________________________________________________________________________________________________________________________________________________________________________________________________________________________________________________________________________________________________________________________________________________________________________________________________________________________________________________________________________________________________________________________________________________________________________________________________________________________________________________________________________________________________________________

18. Is there any additional support that would be helpful to receive?

__________________________________________________________________________________________________________________________________________________________________________________________________________________________________________________________________________________________________________________________________________________________________________________________________________________________________________________________________________________________________________________________________________________________________________________________________________________________________________________________________________________________________________________________________________________________________________________________________
